# CpG-island fragments from the *HNRPA2B1*/*CBX3 *genomic locus reduce silencing and enhance transgene expression from the hCMV promoter/enhancer in mammalian cells

**DOI:** 10.1186/1472-6750-5-17

**Published:** 2005-06-03

**Authors:** Steven Williams, Tracey Mustoe, Tony Mulcahy, Mark Griffiths, David Simpson, Michael Antoniou, Alistair Irvine, Andrew Mountain, Robert Crombie

**Affiliations:** 1 ML Laboratories PLC-Research Division, MED IC4, Keele University Science and Business Park, Keele, Staffordshire, ST5 5SP, UK; 2 Nuclear Biology Group, Division of Medical and Molecular Genetics, GKT School of Medicine, Kings College London, Guy's Campus, London Bridge, London, SE1 9RT, UK

## Abstract

**Background:**

The *hCMV *promoter is very commonly used for high level expression of transgenes in mammalian cells, but its utility is hindered by transcriptional silencing. Large genomic fragments incorporating the CpG island region of the *HNRPA2B1 *locus are resistant to transcriptional silencing.

**Results:**

In this report we describe studies on the use of a novel series of vectors combining the *HNRPA2B1 *CpG island with the hCMV promoter for expression of transgenes in CHO-K1 cells. We show that the CpG island gives at least twenty-fold increases in the levels of EGFP and EPO observed in pools of transfectants, and that transgene expression levels remain high in such pools for more than 100 generations. These novel vectors also allow facile isolation of clonal CHO-K1 cell lines showing stable, high-level transgene expression.

**Conclusion:**

Vectors incorporating the *hnRPA2B1 *CpG island give major benefits in transgene expression from the *hCMV *promoter, including substantial improvements in the level and stability of expression. The utility of these vectors for the improved production of recombinant proteins in CHO cells has been demonstrated.

## Background

Despite recent progress in elucidating the molecular basis of gene expression the practicality of achieving reliable, stable, high-level transgene expression in mammalian cells remains a major challenge. This inefficiency of transgene expression is largely attributable to transcriptional silencing, which typically involves methylation at CpG DNA sequences, histone deacetylation and chromatin condensation in the vicinity of the integration site [1,2]. When exogenous genes are introduced into cultured cells, many of the integration events lead to rapid transgene silencing, whilst the remainder give widely varying expression levels [3,4]. For this reason the use of cultured mammalian cells to produce recombinant proteins (except at very small scale) usually requires labour-intensive isolation of rare, clonal cell lines that stably express the transgene at high levels. Similarly, chromatin structure-related silencing and extreme variability in expression hinder the use of transgenic animals for purposes such as defining novel gene function and manufacture of therapeutic proteins [5,6]. Furthermore, achieving clinical utility with gene therapies (especially for chronic diseases), is greatly hindered by inadequate levels and duration of therapeutic transgene expression, resulting partly from transcriptional silencing [7,8]. The identification of elements capable of maintaining a transcriptionally competent ("open") chromatin domain resistant to silencing, irrespective of tissue type or integration site, is therefore an important objective in the development of technology giving more efficient transgene expression in mammalian cells, for many important applications.

We have reported previously that large DNA fragments containing CpG islands from the human *TBP*-*PSMB1 *and *HNRPA2B1-CBX3 *loci (designated *TBP *and *RNP *below) are resistant to heterochromatin-mediated silencing of these genes [9]. These regions are structurally similar, containing dual divergently transcribed promoters embedded within an extended methylation-free CpG island. The divergent promoters drive expression of ubiquitously expressed housekeeping genes, encoding the TATA binding protein and proteasome component-B1 in the case of the *TBP *locus, and the *heterogeneous ribonucleoprotein A2/B1 *and *chromobox homolog 3 *in the case of the *RNP *locus. Our previous studies showed that with these large CpG island-containing fragments, active transcription from the endogenous promoters was maintained even upon integration into centromeric heterochromatin, that single copy integration at different sites gave very similar levels of gene expression, and that position effect variegation was greatly reduced [9].

The endogenous promoters of the housekeeping genes at the *TBP *and *RNP *loci are relatively weak. In this paper we report studies with a series of novel expression vectors in which the CpG island from the *RNP *locus was combined with the immediate early promoter/enhancer of the human cytomegalovirus (*hCMV*), the promoter most commonly used to achieve strong, non-selective expression in mammalian cells for many applications. We show that the *RNP *CpG island confers major benefits in transgene expression from this promoter, including substantial improvements in the level of expression and proportion of transfected cells that express at detectable levels. We show that with these novel vectors expression remains high on prolonged subculturing. We also demonstrate the utility of these vectors for the improved production of recombinant proteins in Chinese Hamster Ovary (CHO) cells.

## Results and Discussion

### *RNP *CpG island fragments enhance transgene expression and confer resistance to silencing when linked to the *hCMV *promoter

**Figure 1:**
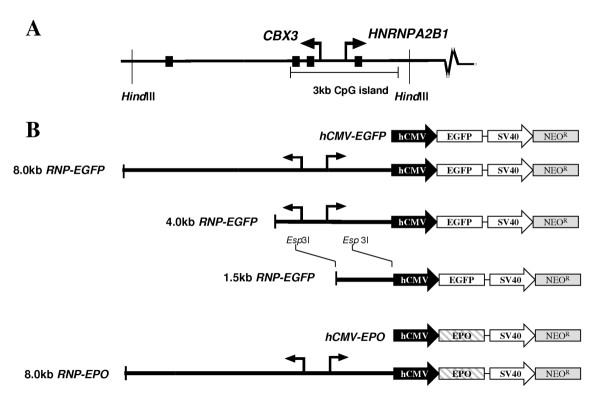
The human *HNRPA2B1/CBX3 *(*RNP*) locus and constructs used in EGFP and EPO expression studies. **A. **Organisation of the *RNP *genomic locus. The figure indicates the locations of the divergent promoters (arrows), the exons (black boxes) and the 8.0 kb Hind *III *fragment that includes the 3 kb unmethylated CpG island. **B. ***EGFP *and *EPO *expression constructs. All constructs carried the Neo^r ^gene preceded by the *SV40 *promoter for selection of stable transfectants of CHO-K1 cells.

Our previous report concerned the ability of large DNA fragments containing the *RNP *CpG island to inhibit transcriptional silencing of genes expressed from the promoters of the endogenous housekeeping genes [9]. With a view to developing vectors allowing facile generation of constructs for improved, high-level transgene expression, we first evaluated smaller fragments containing this CpG island. As shown in Figure 1A the *HNRPA2B1 *and *CBX3 *genes are divergently transcribed from two separate promoters embedded within a methylation-free CpG island approximately 3 kb in length [9]. We evaluated three CpG island-containing fragments of length 8.0 kb, 4.0 kb and 1.5 kb (Figure 1B). All three fragments contained the dual divergent promoters. The 8.0 kb and 4.0 kb fragments both contained the entire methylation-free CpG island, while the 1.5 kb fragment contained approximately half of this region. These fragments were incorporated into vectors for expression of EGFP from the *hCMV *promoter as indicated in Figure 1B.

**Figure 2:**
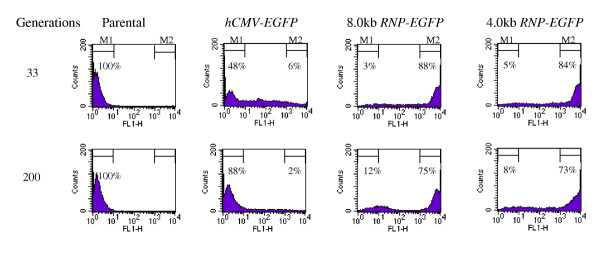
FACScan analysis of EGFP expressing CHO-K1 cells. Following stable transfection with *hCMV-EGFP*, 8.0 kb *RNP-EGFP*, or 4.0 kb *RNP-EGFP*, pools of >100 transfectants were cultured continuously with drug selection for 200 generations, and FACScan analysis conducted after 33 and 200 generations. The results are plotted as green fluorescence on a logarithmic scale (FL1-H) versus number of events on a linear scale (counts). Figures for gate M1 are % cells with undetectable fluorescence; those for gate M2 are % cells showing greater than 1000 fluorescence units.

Following stable transfection of these constructs into CHO-K1 cells, *EGFP *expression was quantified by FACScan analysis of transfectant pools after 33 and 200 generations of culture (Figure 2). After 33 generations pools derived with *hCMV-EGFP *showed a characteristically wide range of expression levels, manifest as a broad plateau rather than a discreet peak of EGFP expressing cells. Interestingly, 48% of the cells showed no detectable expression and only 6% of the cells showed expression exceeding 1000 fluorescence units at this stage of culture. By contrast, after 33 generations of culture for the 8.0 and 4.0 kb *RNP-EGFP *containing populations only 3% and 5% of the cells respectively showed no detectable expression, with 88% and 84% of the cells respectively showing median expression exceeding 1000 fluorescence units. The majority of cells in these populations showed very high expression levels, as evidenced by the steep shoulder at the end of the detectable range. Similarly, after 200 generations of culture these stably transfected cell populations contained 75% and 73% of cells with median fluorescence exceeding 1000 units. At the same stage of culture only 2% of cells in the pool transfected with *hCMV-EGFP *showed fluorescence exceeding 1000 units.

**Figure 3:**
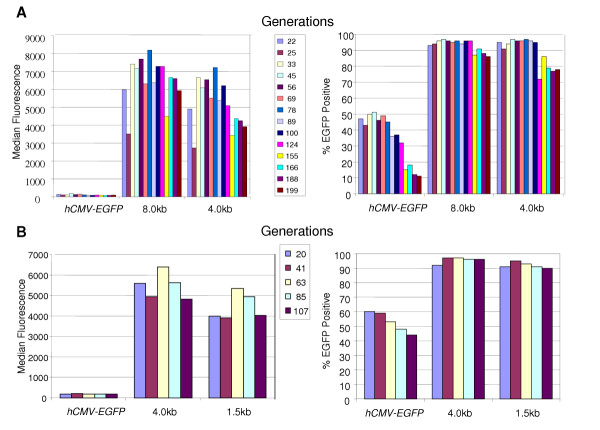
*RNP *CpG island fragments enhance the level and stability of EGFP expression in stably transfected CHO-K1 cells. **A. **Histograms representing the median fluorescence and percentage cells with detectable EGFP expression for CHO-K1 cells stably transfected with *hCMV-EGFP*, 8.0 kb *RNP-EGFP *or 4.0 kb *RNP-EGFP*. Following transfection pools of >100 transfectants were cultured continuously with drug selection for 107 generations and FACScan analysis conducted every few days. **B. **Histograms representing the median fluorescence and percentage cells with detectable EGFP expression for CHO-K1 cells stably transfected with *hCMV-EGFP*, 4.0 kb *RNP-EGFP *or 1.5 kb *RNP-EGFP*. Following transfection pools of >100 transfectants were cultured continuously with drug selection for 107 generations and FACScan analysis conducted every few days

Figure 3 (A and B) shows the results (in histogram format) of similar experiments, conducted over 199 or 107 generations of culture, with vectors containing the 8.0, 4.0 and 1.5 kb CpG islands. The 1.5 kb *RNP *CpG island fragment conferred improvements in level of expression from the *hCMV *promoter and percentage of cells showing detectable *EGFP *expression that were very similar to those observed for the larger fragments. FACScan analysis also showed the characteristic steep shoulder associated with the larger *RNP *fragments (data not shown). Overall, the 8.0, 4.0 and 1.5 kb *RNP *fragments reproducibly gave 20- to 40-fold increases in the median expression levels of EGFP compared to that observed for the *hCMV *promoter alone, and these increased levels were maintained through at least 107 generations of continuous culture.

**Figure 4:**
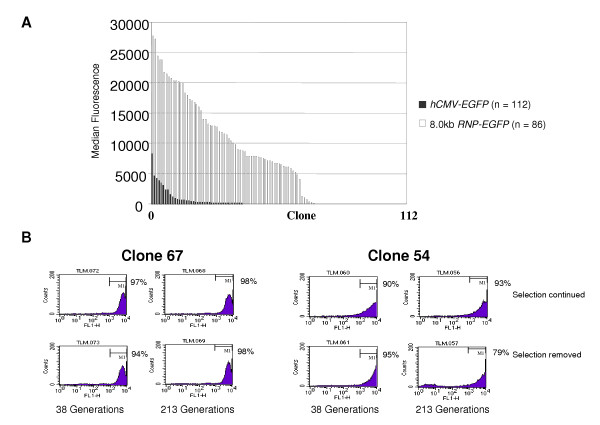
The 8.0 kb *RNP *CpG island fragment gives a high proportion of clonal CHO-K1 cell lines showing high-level, stable expression of EGFP. Following stable transfection of CHO-K1 cells clonal lines were derived by limiting dilution. Median fluorescence levels were measured for each clone by FACScan analysis after 22 generations of culturing with drug selection. **A**. Histograms representing the median fluorescence levels for clones generated with *hCMV-EGFP *(n = 112) and 8.0 kb RNP-EGFP (n = 86). **B**. FACscan analysis of two clonal lines described in A. Clonal cell lines were subcultured continuously with or without drug selection for a further 213 generations, with analysis after 38 and 213 generations. Figures for gate M1 are % cells showing greater than 1000 fluorescence units.

The effects of the CpG island fragments on EGFP expression level and stability were further studied using clonal CHO-K1 cell lines rather than transfected pools. Figure 4A shows the results of quantifying EGFP expression for clones isolated following stable transfection with the *hCMV-EGFP *and 8.0 kb *RNP-EGFP *vectors. Of more than a hundred clonal lines containing the *hCMV-EGFP *construct the majority (60 of 112) showed no detectable EGFP expression, with only a single clone displaying a median expression level that exceeded 5000 fluorescence units. In contrast, for clones generated with the 8.0 kb *RNP-EGFP *construct, the great majority (74 of 86) did show detectable EGFP expression and a large proportion (63 of 86) had a median expression level exceeding 5000 units. 41 of 86 clones with the 8.0 kb RNP-EGFP construct showed expression exceeding that of the best *hCMV-EGFP *clone. Of the 12 clones generated with this construct that showed no detectable EGFP expression, all those examined by Southern blot analysis proved to have deletions extending (at least) into the RNP sequences (data not shown). These deletions presumably occurred prior to or during the integration process. Similar analysis of non-expressing clones generated with the *hCMV-EGFP* construct revealed many with no detectable deletion. These results suggest that the 8.0 kb *RNP* fragment confers a substantial increase in the proportion of integration events that lead to detectable gene expression.

Ideally, for large-scale manufacture of protein therapeutics clonal cell lines producing the proteins are expanded without drug selection from a master cell bank. Where proteins are manufactured for use in humans the regulatory authorities require evidence that the manufacturing process gives production that is stable (i.e that falls within defined specification limits) over 25 to 30 generations of culture. To investigate the suitability of vectors containing the *RNP *CpG island fragments for this purpose, four clonal lines generated with the 8.0 kb *RNP-E*GFP construct and showing median expression that exceeded 5000 fluorescence units after 118 generations of culture in the presence of G418, were cultured with and without drug selection for a further 213 generations. For all four clones no reduction was observed in the percentage of cells showing expression exceeding 1000 fluorescence units after 213 generations of culture with drug selection. Also for all four clones no reduction was observed in the percentage of such cells after 38 generations without drug selection. Slight instability of expression was observed for one clone, #54, on very prolonged culture without drug selection. As shown in Figure 4B for this clone the percentage of cells showing expression exceeding 1000 units declined from 95% to 79% between 38 and 213 generations, while for the other three clones, exemplified by #67, no such decline was observed.

### *RNP *CpG island vectors give improved yields of recombinant proteins in CHO cells

**Figure 5:**
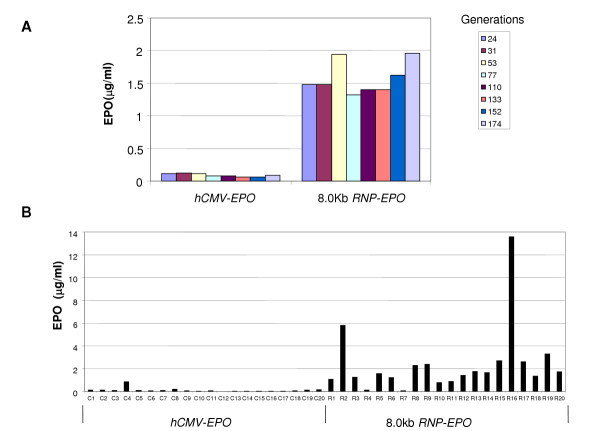
The 8.0 kb *RNP *fragment increases the yield of EPO in stably transfected CHO-K1 cells. A EPO production from pools of CHO-K1 cells stably transfected with *hCMV-EPO *or 8.0 kb *RNP-EPO*. Following selection on G418 pools of transfectants were cultured continuously with drug selection for 174 generations. Assays for EPO production were conducted every few days. B EPO production from clonal CHO-K1 cell lines derived with *hCMV-EPO *or 8.0 kb *RNP-EPO*. Clonal lines were derived by limiting dilution from pools of transfectants selected on G418. Colonies were expanded for 35 generations with drug selection prior to assay for EPO production.

The utility of the *RNP *CpG island vectors for recombinant protein production was further examined using the *erythropoietin *(*EPO*) gene. Constructs were generated with the *EPO *cDNA sequence under the control of either the *hCMV *promoter alone or in combination with the 8.0 kb *RNP *fragment (Figure 1B). Following stable transfection into CHO-K1 cells EPO production was measured for pools of transfectants over 174 generations of culture (Figure 5A). The 8.0 kb *RNP-EPO *construct gave a substantially higher yield of EPO at all time points than that observed with *hCMV-EPO*. On average the *RNP *CpG island vector gave a twenty-fold improved yield of EPO, and the improved production was maintained through at least 100 generations of subculture.

Clonal CHO-K1 lines were derived from transfectants generated with the *hCMV-EPO *and 8.0 kb *RNP-EPO *expression constructs. Twenty clones were chosen at random and assessed for EPO production (Figure 5B). For clones derived with *hCMV-EPO*, 6/20 produced no detectable EPO and only 1/20 (C4) showed a yield (0.85 μg/ml) exceeding 0.5 μg/ml. In marked contrast, 20/20 clones harbouring 8.0 kb *RNP-EPO *showed detectable EPO production, 18/20 produced more than 0.5 μg/ml, 16/20 produced more than 1 μg/ml, and one clone (R16) produced almost 14 μg/ml. Thus inclusion of the 8.0 kb *RNP *fragment in the expression construct resulted in the facile isolation of clones with improved EPO production, the average yield for the twenty clones being twenty-one-fold higher than with *hCMV-EPO*, and the best producing clone with 8.0 kb *RNP-EPO *giving sixteen-fold higher productivity than C4, the highest producing *hCMV-EPO *line.

### Increased expression with RNP CpG island vectors is not due to increased transgene copy number

The possibility that CpG island vectors give improved transgene expression through integration at increased copy number was evaluated by conducting copy number analysis on genomic DNA for clonal lines expressing EGFP following transfection with *hCMV-EGFP *(13 lines) or 8.0 kb *RNP-EGFP *(9 lines). All these lines proved to have copy numbers of two or three. The highest expressing clones with both constructs had a copy number of three, but so did some of the lowest expressing clones for both constructs. Overall, statistical analysis of variance (by the ANOVA single factor method) indicated no significant correlation between level of expression and copy number for either construct.

## Conclusion

The *hCMV *promoter has been by far the most commonly used promoter for high level transgene expression in mammalian cells, for a wide range of applications that includes the production of protein therapeutics in cultured cells, transgenic animals and gene therapies. Its utility in expressing transgenes for all these applications, however, has been hindered by its susceptibility to silencing, largely through effects involving adverse chromatin structure. For example, the isolation of clonal cell lines or transgenic animals showing stable, high level expression of transgenes is usually a slow and labour-intensive procedure because most integration events lead to silencing.

The results we report here show that incorporating a *RNP *CpG island fragment immediately upstream of the *hCMV *promoter gives major benefits in expression from the latter. These benefits include a substantial increase in the median level of expression observed in pools of transfectants, together with a substantial improvement in the proportion of cells in the pool that express. The CpG island fragments reproducibly gave 20 to 40-fold increases in the level of expression observed. With both EGFP and EPO these dramatically increased expression levels were maintained through at least 100 generations of subculture. The CpG island fragments also enabled the rapid and facile isolation of clonal cell lines showing stable, high level expression of EGFP and EPO.

Our previous work [9] showed that a 16 kb DNA fragment containing the *RNP* CpG island conferred resistance to heterochromatin-mediated silencing of expression from the endogenous *RNP* promoter and reduced position effect variegation, giving very consistent expression levels in tissue culture cells. In the studies we report here, constructs with the 8.0 kb *RNP* CpG island fragment preceding the *hCMV* promoter gave detectable levels of transgene expression for all integration events in which the construct remained intact, together with substantial increases in the level of expression from the *hCMV* promoter. Analysis of variance (by the single factor ANOVA method) showed these increases to be statistically significant (p = 8.13E-27). These constructs, however, gave much greater variability in levels of expression than those observed in our previous work with a larger CpG island fragment and the endogenous *RNP* promoter. It is not clear whether the greater variability in expression level for the CpG island constructs in the studies we report here than our previous ones is due to the use of a different promoter or different cell line, or occurs because sequences giving more complete isolation from the effects of chromatin structure or transcriptional activity adjacent to the integration site are present on the 16 kb fragment but not on the 8 kb fragment.

The present results demonstrate two very important applications of this novel transgene expression technology. One is the rapid production of recombinant proteins, in the quantities required for basic research, drug discovery and preclinical studies, using stable pools of transfectants. The CpG island technology circumvents the need for slow, labour intensive screening of clonal lines for this purpose. The other is faster and easier identification of clonal cell lines that show stable, high level production as candidates for large scale manufacture of protein therapeutics. With the CpG island technology screening of only 20 clonal CHO cell lines was sufficient to identify lines showing very high yields of EPO, the latter being a blockbuster protein drug that is widely used to treat anaemia associated with renal failure and chemotherapy. It is noteworthy that the *hCMV *and CHO cells used in these experiments are the promoter and cells most commonly used for commercial manufacture of protein therapeutics.

Through their capacity to confer an increase in the proportion of integration events that are productive, together with improvements in the level of transgene expression, *RNP *CpG island vectors have many other potential applications, including use in mammalian cell-based *in vitro *screens for drug discovery, transgenic animals for basic research and drug discovery, and gene therapy. For some of these applications, notably those involving transgene delivery with integrating viral vectors, gene expression elements of small size are required. The data we present here suggest that the 1.5 kb *RNP *fragment confers major benefits in expression level from the *hCMV *promoter. Efforts to further characterise and minimise these elements are ongoing, but the 1.5 kb fragment should be small enough for incorporation into most viral vectors.

The mechanism by which the *RNP *CpG island reduces silencing and improves transgene expression is under investigation in our laboratories. The data we report here show that the mechanism does not involve increasing the copy number of the integrated transgene. Our results to date, reported previously [9] and in this paper, are consistent with these elements being able to establish and maintain a more open chromatin domain irrespective of the local chromosome environment. We propose that the promoter-containing CpG-islands of housekeeping genes possess a chromatin remodelling function and that this is designated a "Ubiquitously-acting Chromatin Opening Element", or "UCOE".

## Methods

### Expression vectors

*hCMV-EGFP *was vector *pEGFP-N1 *(Clontech, Cambridge, UK), which contains the *hCMV *promoter/enhancer on a 589 bp fragment. *RNP *CpG island-containing vectors were constructed by inserting genomic fragments from the RNP locus into the blunted Ase *I *site of *hCMV-EGFP*. These fragments were blunted versions of the 8.0 kb Hind *III *fragment, 4.0 kb BamH *I*-Hind *III *fragment and 1.5 kb Esp3*I *fragments to give 8.0 kb *RNP-EGFP*, 4.0 kb *RNP-EGFP *and 1.5 kb *RNP-EGFP *respectively (Figure 1B).

The *erythropoietin *(*EPO*) cDNA was isolated by PCR amplification from a Quick Clone Foetal Liver cDNA library (Clontech, Cambridge, UK). The resulting 705 bp product was subcloned using the TA-cloning vector *pCR3*.1 (Invitrogen, Paisley, UK) generating *pCR-EPO*. *hCMV-EPO *was constructed by subcloning an NheI-NotI fragment from *pCR-EPO *into the respective sites within the vector *pEGFP-NI*. 8.0 kb *RNP-EPO *was constructed by subcloning the blunt-ended 8.0 kb Hind *III *fragment into the blunted Ase *I *site of *hCMV-EPO *(Figure 1B).

### Cell lines and transfections

CHO-K1 cells were grown in HAMS F12 (Invitrogen, Paisley, UK) plus 4500 mg/l L-ananyl-L-glutamine, 10 μg/ml each of penicillin and streptomycin, and 10% (v/v) heat inactivated foetal calf serum (FCS; Invitrogen, Paisley, UK). Transfection was carried out by electroporation using approximately 10^7 ^cells from 80% confluent cultures and a BioRad Gene Pulser II ™ set to deliver a single pulse of 975 μF at 250 V. Transfections used 1 μg of linearised *hCMV *plasmid and equivalent molar quantities for expression vectors of different size. Stably transfected cells were selected and maintained in growth medium containing 400 μg/ml geneticin sulphate (G418; Sigma, Poole, UK). Clonal cell lines were derived from stable transfected pools by standard limiting dilution techniques.

### Quantification of transgene expression

Analysis of cells transfected with EGFP reporter constructs was with a Becton-Dickinson FACScan using the parental CHO-K1 cell line as a background, autofluorescence control. For EPO quantitation cells were seeded at 10^6 ^cells/well in 6-well plates and incubated in FCS-containing medium over 48 hours. Conditioned medium samples were assayed for EPO using a Quantikine IVD ELISA kit (R&D Systems Europe, Abingdon, UK)

### Transgene copy number analysis

Copy number was determined by quantitative PCR (QPCR) using oligonucleotide primers and Beacon probes designed using Beacon Designer v2.0 software (Premier Biosoft International, CA, USA) specific to *hCMV *or *EGFP *sequences and to the endogenous housekeeping gene β*-actin *as a copy number control. Multiplex QPCR reactions containing optimised oligonucleotide and probe concentrations and ~500 ng of template DNA were performed on the MX4000™ multiplex PCR quantitative PCR system according to the manufacturers protocol (Stragene, La Jolla, CA), and subsequent data manipulations performed using the MX4000™ analysis software (Stratagene, La Jolla, CA). Copy number was assigned by comparison of the *hCMV *and/or *EGFP*: β*-actin *endpoint fluorescence ratio with that of a verified single copy integrant.

## Authors' contributions

SW carried out vector construction, EGFP pool transfection and analysis and experimental design.

TM and TM participated in vector construction, CHO-KI transfection, EGFP pool and clonal analysis.

MG carried out EPO cloning, CHO-KI pool and clonal analysis.

DS carried out copy number analysis and manuscript preparation.

MA was the original investigator and participated in the conception of the study.

AI participated in coordination of the project and manuscript preparation.

AM and RC were involved in the conception, experimental design and manuscript preparation.
